# 177. Infectious Diseases Consultation Associated with Decreased Mortality in Gram-Negative Bacteremia

**DOI:** 10.1093/ofid/ofad500.250

**Published:** 2023-11-27

**Authors:** Rebecca Bruning, Kyler Runyon, Donna R Burgess, Sarah Cotner, Katie B Olney, Jeremy VanHoose, Hanine El Haddad, David Burgess

**Affiliations:** University of Kentucky HealthCare, Lexington, Kentucky; University of Kentucky HealthCare, Lexington, Kentucky; UK HealthCare, Lexington, KY; UK HealthCare, Lexington, KY; University of Kentucky HealthCare, Lexington, Kentucky; UK HealthCare, Lexington, KY; UT Southwestern Medical Center, Not Hispanic or Latino, KY; UK HealthCare, Lexington, KY

## Abstract

**Background:**

Bloodstream infections (BSIs) are major causes of morbidity and mortality for which prior analyses and validated scoring tools have attempted to stratify risk. This study investigated institutional and epidemiologic predictors of mortality with Gram-negative BSIs to better identify targeted antimicrobial stewardship efforts to improve outcomes.

**Methods:**

All patients with aerobic, monomicrobial, Gram-negative BSIs admitted and discharged from our academic medical institution between July 2022 and March 2023 were included. Data collected included patient demographics, microbiological data, clinical management, length of stay, ID consultation, and clinical outcomes (in-hospital or hospice mortality). Univariate and multivariable logistic regression models were performed to identify variables that were primary drivers for inpatient mortality.

**Results:**

Overall, 259 patients (83.7% Caucasian, 49% male, mean age 53.7 yrs) were identified. The most common organisms were *E. coli* (37.8%), *K. pneumoniae* (11.2%), *P. aeruginosa* (10.0%), *S. marcescens* (8.5%), and *E. cloacae* (6.2%). The majority (65.3%) were community acquired infections with 41.7% in the ICU, and 46.7% received an infectious diseases consult. The overall mortality was 14.7% with the highest mortality due to *P. aeruginosa* (34.6%) followed by *S. marcescens* (27.3%), *K. pneumoniae* (13.8%), *E. cloacae* (12.5%), and *E. coli* (8.2%). Overall mortality was significantly associated with several parameters in a univariate analysis (Table 1). In multivariable regression analysis hospital-acquired infection (0=0.002), cancer (p=0.012), qPitt > 2 (p< 0.001), SBP< 100 or vasopressor use at 72-96 hrs after positive blood culture (p=0.017) was associated with increased risk of mortality. The only parameter to reduce mortality within the multivariable regression analysis, by more than two-fold, was ID consultation (p=0.022).

Parameters Associated with Mortality in Univariate Analysis

**Figure:**
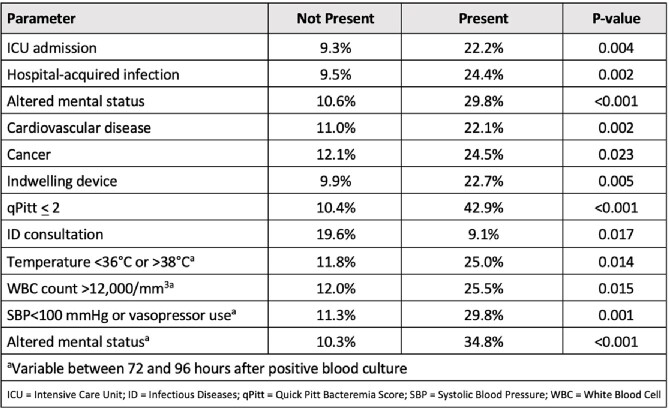

**Conclusion:**

The only factor found to significantly reduce mortality was formal ID consultation. ID consultation should be considered part of routine care for all patients with Gram-negative bacteremia.

**Disclosures:**

**Katie B. Olney, PharmD, BCIDP**, The Society of Infectious Diseases Pharmacists (SIDP): Grant/Research Support

